# Bismuth incorporation and the role of ordering in GaAsBi/GaAs structures

**DOI:** 10.1186/1556-276X-9-23

**Published:** 2014-01-13

**Authors:** Daniel F Reyes, Faebian Bastiman, Chris J Hunter, David L Sales, Ana M Sanchez, John P R David, David González

**Affiliations:** 1 Departamento de Ciencia de los Materiales e IM y QI, Universidad de Cádiz, Puerto Real, Cádiz 11510, Spain; 2 Department of Electronic and Electrical Engineering, The University of Sheffield, Mappin Street, Sheffield S1 3JD, UK; 3 Physics Department, University of Warwick, Coventry CV4 7AL, UK

**Keywords:** GaAsBi/GaAs epilayers, Molecular beam epitaxy, Bi composition, CuPt_B_-type ordering

## Abstract

The structure and composition of single GaAsBi/GaAs epilayers grown by molecular beam epitaxy were investigated by optical and transmission electron microscopy techniques. Firstly, the GaAsBi layers exhibit two distinct regions and a varying Bi composition profile in the growth direction. In the lower (25 nm) region, the Bi content decays exponentially from an initial maximum value, while the upper region comprises an almost constant Bi content until the end of the layer. Secondly, despite the relatively low Bi content, CuPt_B_-type ordering was observed both in electron diffraction patterns and in fast Fourier transform reconstructions from high-resolution transmission electron microscopy images. The estimation of the long-range ordering parameter and the development of ordering maps by using geometrical phase algorithms indicate a direct connection between the solubility of Bi and the amount of ordering. The occurrence of both phase separation and atomic ordering has a significant effect on the optical properties of these layers.

**PACS:**

78.55.Cr III-V semiconductors; 68.55.Nq composition and phase identification; 68.55.Ln defects and impurities: doping, implantation, distribution, concentration, etc; 64.75.St phase separation and segregation in

## Background

The development of new semiconductor materials with dilute bismuth (Bi) has aroused great interest among researchers in the recent years. GaAsBi exhibits a band gap reduction of up to 90 meV/% Bi, a strong enhancement of spin-orbit splitting and a temperature-insensitive band gap [1-3] which are attractive properties for infrared lasers, photodetectors and terahertz optoelectronic applications. Certainly, compositions from 6% to 11% in bulk GaAsBi epilayers cover the important telecommunication band (1.2 to 1.55 μm) [4,5]. However, the growth of even low Bi content III-V alloys has been hindered by a large miscibility gap and a very small equilibrium solid solubility. Attempting to add a larger group V solute atom (like Bi) into a solvent material (like GaAs) leads to an increase in the substitutional energy owing to the large atomic size difference and, as a consequence, a reduction of the solubility of the solute atom [6]. Growth temperatures below approximately 400°C enhance solubility; however, the quality of GaAsBi is highly dependent on the Bi composition and the growth temperature. As a consequence, the limited solubility exhibited by GaAsBi has also been shown to lead to alloy clustering and phase separation, even for low Bi contents [7].

On the other hand, it is well known that CuPt_B_ atomic order mainly occurs in ternary alloys near the commensurable composition of *x* ≈ 0.5, and indeed, it is frequently observed for III-V ternary semiconductor compounds close to this composition [8]. However, several studies showed that III-V alloys with dilute Bi exhibited CuPt_B_-type ordering, despite a relatively low Bi content [7,9]. Zhang et al. [6] suggested that when a (2 × 1) surface reconstruction is present on the (001) surface during growth, an increase in solubility is achieved. Strain energy is reduced by incorporating smaller atoms into the atomic positions under compression and larger atoms in atomic positions under tension leading to an ordered structure. Bi could act in a similar way occupying positions under tension, enabling the incorporation of a larger Bi content than would be expected in a random alloy. Nevertheless, the formation of CuPt_B_-type ordering can produce changes in the crystal structure [8], modifying the band gap [10,11] and valence band splitting [12]. Characterizing and correlating CuPt_B_-type ordering with the electronic and optical properties of GaAsBi alloys are necessary in order to understand the properties of this atypical alloy.

The present work analyses the Bi incorporation in GaAs_1−*x*_Bi_*x*_/GaAs(100) epilayers grown by molecular beam epitaxy (MBE) using advanced analytical transmission electron microscopy (TEM) and photoluminescence (PL) techniques. The relationship between the inhomogeneous Bi composition and the presence of CuPt_B_ ordering is presented. High-resolution TEM (HRTEM) is used to render ordering maps and provide an estimate of the long-range order (LRO) parameter (*S*). The aim of this work was to provide a useful tool to determinate the distribution of ordering and characterize the quality of GaAsBi nanostructures.

## Methods

### Equipment and techniques

The analysed samples were grown by solid source MBE. The samples comprise a 500-nm GaAs buffer grown at 580°C, followed by either a 25-nm (sample S25) or a 100-nm (sample S100) GaAsBi layer grown at approximately 380°C ± 10°C. The GaAsBi layers were capped with a 100-nm GaAs layer grown at the GaAsBi growth temperature. An As_4_/Ga/Bi beam equivalent pressure ratio of 40:2:1 and a growth rate of 1.0 μm/h determined from reflection high energy electron diffraction (RHEED) oscillations were used for both samples.

For room-temperature photoluminescence (RT-PL) measurements, the excitation source was a 532-nm diode pumped solid-state laser operating with an excitation power density of 114 Wcm^−2^. The emitted PL was collected by a Cassegrain lens and then focused onto the entrance slit of the monochromator before being detected by a liquid nitrogen cooled germanium detector. A phase-sensitive lock-in detection technique was also used to eliminate the contribution from the background light to the measured PL. Structural and analytical analyses were performed in cross-sectional samples prepared using conventional techniques by transmission electron microscopy. Diffraction contrast imaging and selected area electron diffraction (SAED) patterns were obtained in a JEOL 1200EX (JEOL Ltd, Akishima-shi, Tokyo, Japan) at 120 kV. HRTEM images for fast Fourier transform (FFT) reconstruction were obtained with a JEOL-2100 at 200 kV. Z-contrast high-angle annular dark field (HAADF) in scanning TEM mode and energy-dispersive X-ray (EDX) spectroscopy with an Oxford Inca Energy-200 detector (Oxford Instruments, Abingdon, UK) were performed in a JEOL 2010 at 200 kV. HRTEM images were post-processed for FFT reconstruction and geometrical phase analysis (GPA) by using the GPA software running in a MATLAB routine and Digital Micrograph software (GATAN Inc., Pleasanton, CA, USA).

### Order parameter estimation

The Bragg-Williams LRO parameter (***S***) is used to quantify the degree of ordering across two types of site occupied by atom A and B, *α* − and *β* − sites, respectively. It can be defined as follows [13]:

$$S=\frac{r_{\alpha }-x_{A}}{y_{\beta }}=\frac{r_{\beta }-x_{B}}{y_{\alpha }},$$

where *r*_*α*_ (*r*_*β*_) is the fraction of α-sites (*β*-sites) occupied by the right atom A (B), *x*_A_ (*x*_B_) is the atom fraction of A (B) and *y*_*β*_ (*y*_*α*_) denote the fraction of *β* − sites (*α* − sites). For a completely random crystal, *r*_*α*_ = *x*_A_ and *S* = 0, while for a perfectly ordered structure, *S* = 1. Numerous studies have been conducted to determine the degree of ordering through different techniques, such as nuclear magnetic resonance [14], PL [15] and X-ray diffraction [16]. In X-ray and electron diffraction methods, LRO parameters have been determined from the ratio of superlattice and fundamental reflection intensities weighted by their structure factors by applying kinematical diffraction theory [17].

In general, the electron diffraction method to determine structure factors of alloys does not always allow determination of the LRO parameters because superlattice reflections of ordering alloys are not amenable to critical voltage techniques [18]. Conventional TEM has also been used in this way; however, the weak intensity of extra reflections makes it impossible to carry out a study of image intensity similar to that described by Baxter et al. [19]. To circumvent this, an estimation of the order parameter from the HRTEM images taken at different zones inside the GaAsBi layer was carried out. It is well known that HRTEM images are a two-dimensional intensity pattern produced from a complex interference of the electron beams exiting from the analysed sample. These images carry quantitative information of the sample, namely atomic structure, lattice parameters/strain and chemical information [20]. Furthermore, FFT reconstruction of HRTEM images provides information about the periodicity of the atomic structure which can be correlated to the electron diffraction patterns registered at the back focal plane of the objective lens [21]. In the following, we interpret the bright spots in the FFT images as diffraction spots (reflections) from crystallographic planes of the crystalline phases in the structures. CuPt_B_ ordering in zinc-blende GaAsBi occurs in the alternating {111} planes of group V atoms resulting in a diffraction spot at ½ (111). The intensity of the extra reflections depends on the level of said ordering; hence, the higher the grade of ordering the more intense in the extra reflection in the FFT. Thus, an estimation of ***S*** is given by [22]:

S=ISI11112F111FS,

where *I*_*s*_ and *I*_111_ are the intensity of the ½(111) and (111) spots, respectively; *F*_s_, is the structure factor for a fully ordered alloy and is given by *F*_*s*_ = 2(*f*_As_ − *f*_Bi_) and *F*_111_ = 4(*f*_III_ − *if*_V_) is the structure factor for the {111} reflections.

The absolute diffracted intensity is subject to errors due to several experimental parameters. In order to reduce the error in the measurements, the integrated intensity around the ½(111) and (111) spots is normalized by subtracting the background around the signal obtained close to each diffraction spot, respectively. The FFT method from HREM images, on the other hand, provides LRO parameters in a small selected microscopic area, and therefore, it enables microscopic fluctuations of LRO parameters to be examined.

### Ordering maps from geometric phase algorithm

HRTEM images allow us to extract information on compositional variations and/or the state of deformation of the nanostructures by comparing the actual positions of the unit cells in the image with a reference lattice using such techniques as the peak pairs algorithm or geometric phase analysis [23,24]. Even though these programs are mainly applied to the analysis of the deformation present in the nanostructures, they can be used to perform other types of studies such as the spatial location of different phases and grains [25]. We follow a similar procedure here in order to obtain a spatial map of the distribution of the ordering.

The procedure used for calculating the phase image, the Bragg filtered image and numerical moiré image using the GPA are as described by Hÿtch and co-workers [24,26]. Briefly, the method consists of constructing a differential phase map for a given Bragg region with respect to a reference lattice. In our case, we build numerical moiré images at position *r*, *M*(*r*), by superimposing the real lattice with a reciprocal lattice vector smaller than the average lattice where *M* is a magnification constant as [25,27]:

$$M\left(r\right)=\frac{2\pi g_{r}\cdot r}{M}-2\pi g_{r}\cdot u\left(r\right),$$

where **g**_**r**_ is the reference lattice in reciprocal space and **u**(**r**) is the displacement of the atomic column position from its nominal position. Following this procedure, two translational moiré images (we used *M* = 1) are obtained using **g**_**r**_ as the reference position of each (111) spot in the FFT pattern and a Bragg mask that includes the collinear ½(111) spot associated with the ordering arrangement. The final RGB multilayer reconstructed image is formed from the two inverse FFT (iFFT) images of these selected masks. The spatial localization of ordering in each of the {111} planes is represented in the sets of red and green fringes. In order to improve visualization, a null matrix blue layer is used as background. The red and green fringes in this resultant image are consistent with the presence of ordering where the moiré spacing is proportional to 1/(g − g_r_).

## Results

### Photoluminescence

**Figure 1:**
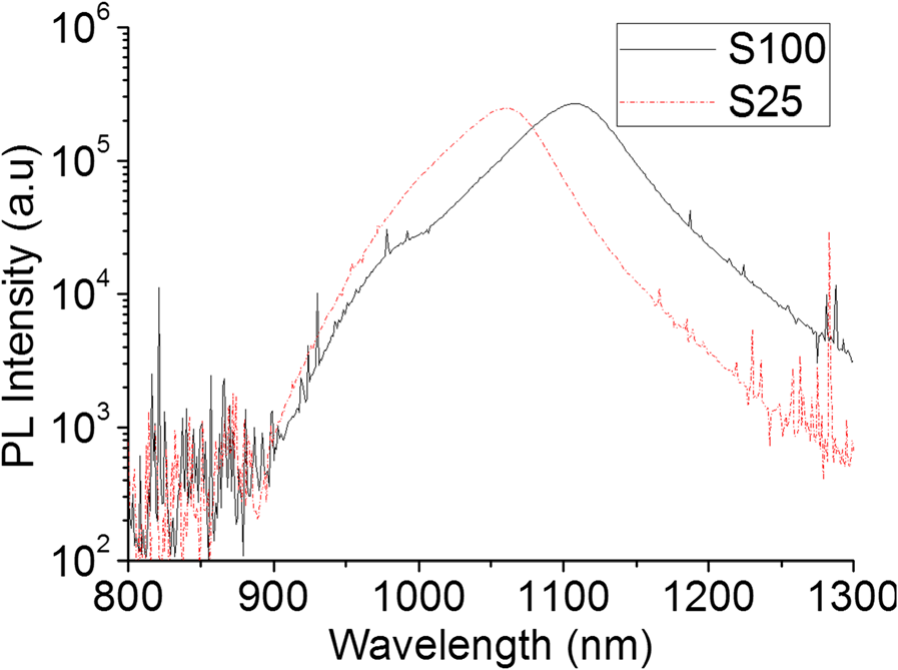
**Room-temperature PL spectra of MBE-grown GaAsBi layers.** S25 (dashed) and S100 (solid) lines.

In order to evaluate the optical emission efficiency, RT-PL measurements were carried out on both samples (Figure 1). Sample S100 showed a bimodal spectrum, with an emission peak at 1,108 nm and a distinct low wavelength shoulder feature at 980 nm. The main peak has a full width at half maximum (FWHM) of 79 meV. However, S25 showed only a single peak centred at 1,057 nm with a FWHM of 75 meV. The PL intensities were nominally identical to within the experimental error.

It is surprising in sample S100 that two distinct PL peaks coexist in a contiguously grown layer, since it is natural for photo-excited carriers to recombine in the deepest well. This indicates that the internal interface between the two GaAsBi regions of different Bi contents is highly perturbed and prevents the free flow of photo-excited carriers.

At RT, the PL emission peaks are dominated by band-to-band transitions, and hence, the PL peak energies can be tentatively correlated to the Bi composition of the material. From the relationship between band gap energy and Bi composition established by Usman et al. [28] the PL peaks of S100 at 1,108 and 980 nm correspond to a Bi content of approximately 5.1% and approximately 2.6%, respectively. Similarly, the main peak of S25 at 1,057 nm corresponds to a Bi content of approximately 4.2%. This indicates that the maximum Bi content of S100 is higher than S25, despite nominally identical flux ratios were used during growth. This discrepancy is believed to be due to an inherent error in the temperature calibration that resulted in S100 being grown approximately 15°C lower than S25 and not a result of the thinner overall layer thickness.

Despite the difference in the absolute peak position, the RT-PL spectra of both samples exhibit a similar envelope comprising (1) a high-wavelength tail and (2) a lower wavelength shoulder. This asymmetric emission indicates that both spectra are formed from the superposition of at least three individual PL peaks. It is therefore possible that the shape of the PL spectra corresponds to structural or compositional features that are present in both samples, whereas the distinct lower energy peak in S100 corresponds to a feature not present in S25.

### Structural and compositional TEM

In order to find an explanation of the PL spectra, TEM studies were carried out by diverse techniques. Low-magnification CTEM images acquired using different diffraction conditions sensitive to defects (not shown in this paper) revealed defect-free epilayers in the electron-transparent area of sample S25 and some isolated dislocations in sample S100. Thus, the RT-PL intensity of both samples is nominally identical despite the presence of threading dislocations in S100; however, their presence at the internal-interface may explain the splitting of the PL peaks in S100. The physical origin of the each of the PL peaks requires further analysis.

**Figure 2:**
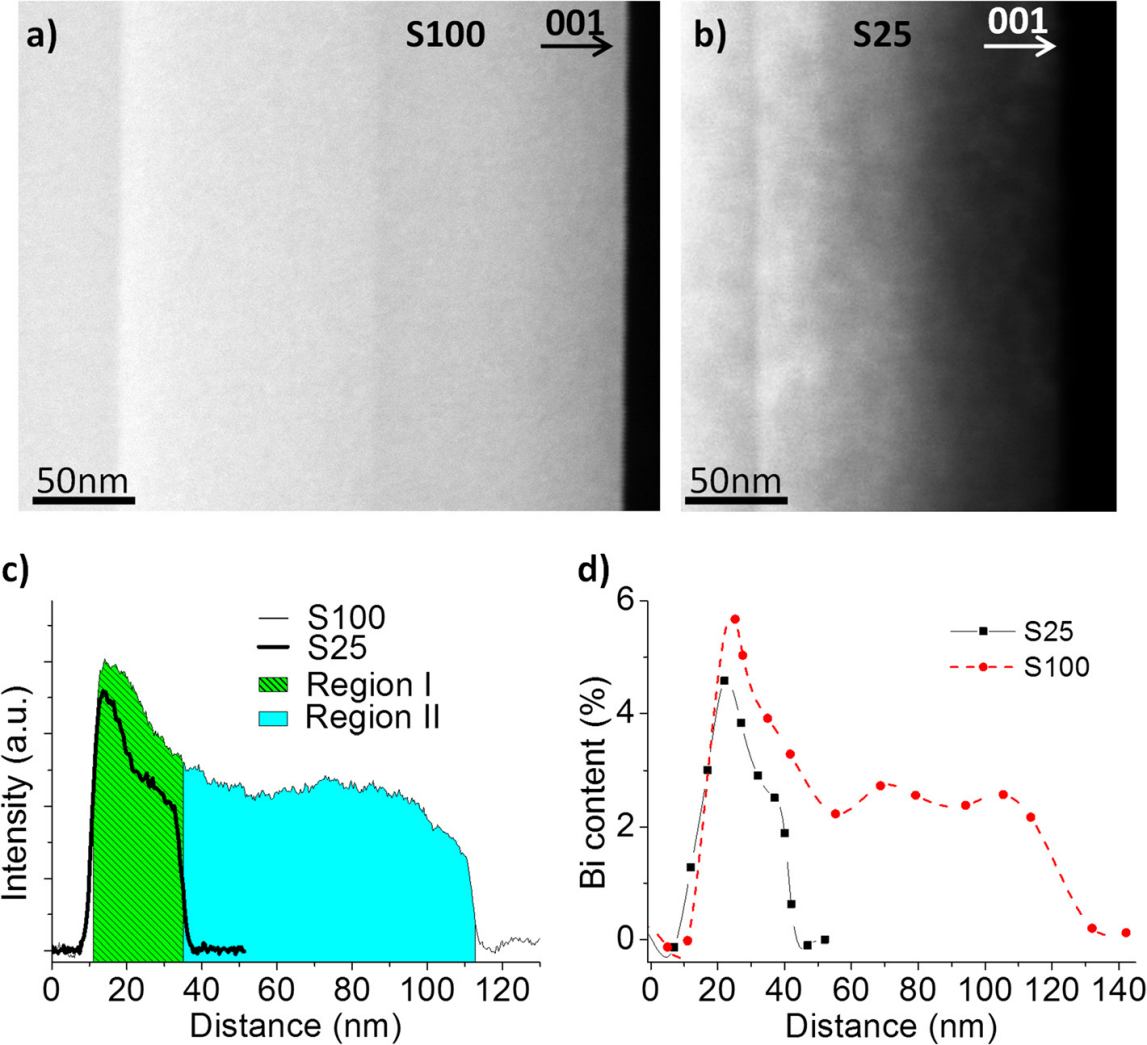
**Compositional distribution in the GaAsBi layers.** HAADF images taken along the [110] pole of samples **(a)** S100 and **(b)** S25. The normalized HAADF intensity profiles **(c)** and point EDX measurements **(d)** performed along the growth direction of both samples, respectively.

HAADF-STEM images were used to study the distribution of bismuth in the GaAsBi layers. Interpretation of this kind of image (also called Z-contrast images) is relatively straightforward, since the contrast is roughly proportional to the square of the atomic number at constant sample thickness [29,30]. Hence, for the case of a ternary alloy where bismuth is the only variable element, brighter contrast should in principle be associated with higher Bi content. Z-contrast images (Figure 2a,b) showed uniform GaAs_1−*x*_Bi_*x*_ layer widths in both samples, corresponding to the nominal ones. Nevertheless, the normalized intensity profiles along the growth direction (Figure 2c) are not constant depicting an inhomogeneous Bi distribution. The image intensity contribution due to sample thickness was subtracted, and the intensity was averaged across more than 100 nm in Figure 2c.

It is possible to distinguish two different regions: (1) the first 25 nm, where from a maximum Bi content an exponential decay of bismuth occurs; and (2) where the Bi content remains almost constant from 25 nm to the end of the layer (i.e. only observable in the case of sample S100). This Bi distribution was confirmed and quantified by EDX analysis. Figure 2d displays the profiles of both samples acquired by point EDX spectra along the growth direction. The EDX spectra show the same tendency observed in the intensity profiles from Z-contrast images and reveal a lower incorporation of Bi in sample S25. The average point EDX spectra measured in the S100 sample reaches a maximum Bi content of 6.1% ± 0.5% at the bottom interfaces that decays to 2.6% ± 0.6% at the top interface. S25 reaches a maximum Bi content of 4.2% ± 0.5%.

All these EDX determined bismuth contents are in reasonable agreement with the composition calculated from the RT-PL spectra. Ascribing individual features of PL spectra to individual components of the highly inhomogeneous layers suggested in Figure 2c are clearly non-trivial. Nevertheless, the correlation of certain physical and PL features is justifiable. Firstly, the main PL peak of both samples seems to correspond to the high Bi content region I. Secondly, the lower energy shoulder present in both samples, but more dominant in S100 seems to correlate with the lower Bi content region. This region is approximately 75 nm thick in S100 compared to <10 nm in S25, thus the dominance of the feature in the spectra of S100 may correspond to the increased region thickness. The exact origin of the high-wavelength tail and the relative intensities of the individual PL emission centres that lead to the superposition spectra require more detailed PL analysis and are the focus of ongoing work.

### Long-range order analysis

**Figure 3:**
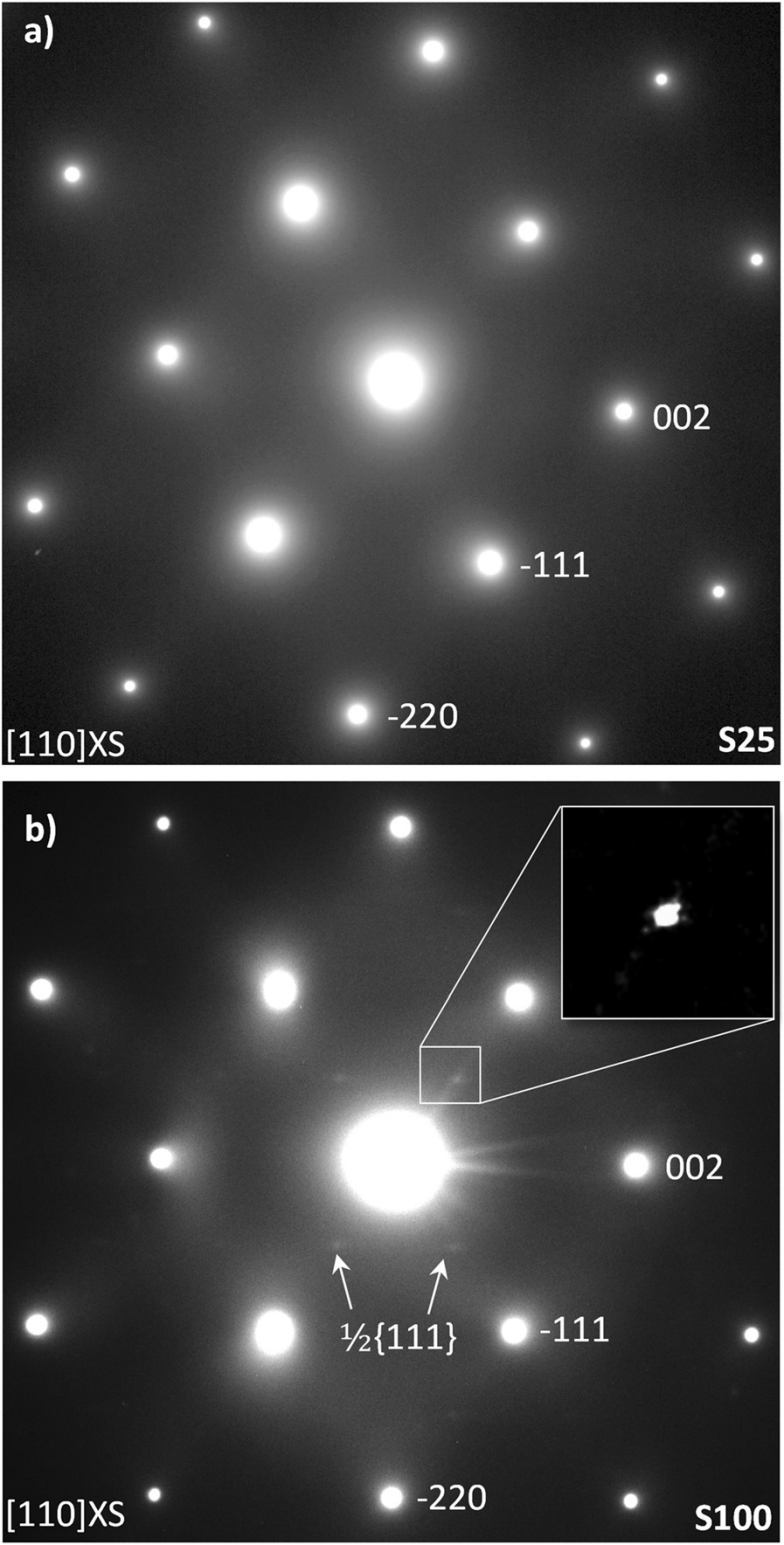
**[110] SAED patterns of samples (a) S25 and (b) S100. (a)** The conventional pattern for the ZB structure, **(b)** the additional ½ 111 superlattice spots associated of a CuPt_B_-type ordering. The inset corresponds with the ½ 111 superlattice spots, magnified and filtered to improve the visualizations.

To date, there has been little work published on the fine microstructural characterization of GaAs_1−*x*_Bi_*x*_ alloys grown by MBE. Certainly, only Norman et al. [7] reported the formation of CuPt-type ordering of the As and Bi atoms on the two {111}_B_ planes for alloy compositions with up to 10% Bi. To investigate the ordering arrangement, cross-sectional TEM samples were prepared along both [110] and [−110] directions, and SAED patterns were taken from the GaAs/GaAsBi/GaAs interfaces. The SAED patterns acquired along the [110] pole exhibit the conventional pattern for the zinc-blende structure. Additional, weak ½ 111-type superlattice spots associated with CuPt_B_-type ordering of the As and Bi atoms on the {111}_B_ planes were found in S100 (Figure 3b) but not in S25 (Figure 3a). The absence of ½ 111-type superlattice spots in the [−110] SAED patterns of both samples (not shown here) indicates a lack of ordering on the {111}_A_ planes. We associate the absence of extra spots in S25 sample to the smaller size of the layer, which could lead to a reduction of its intensity beyond detectable limits.

**Figure 4:**
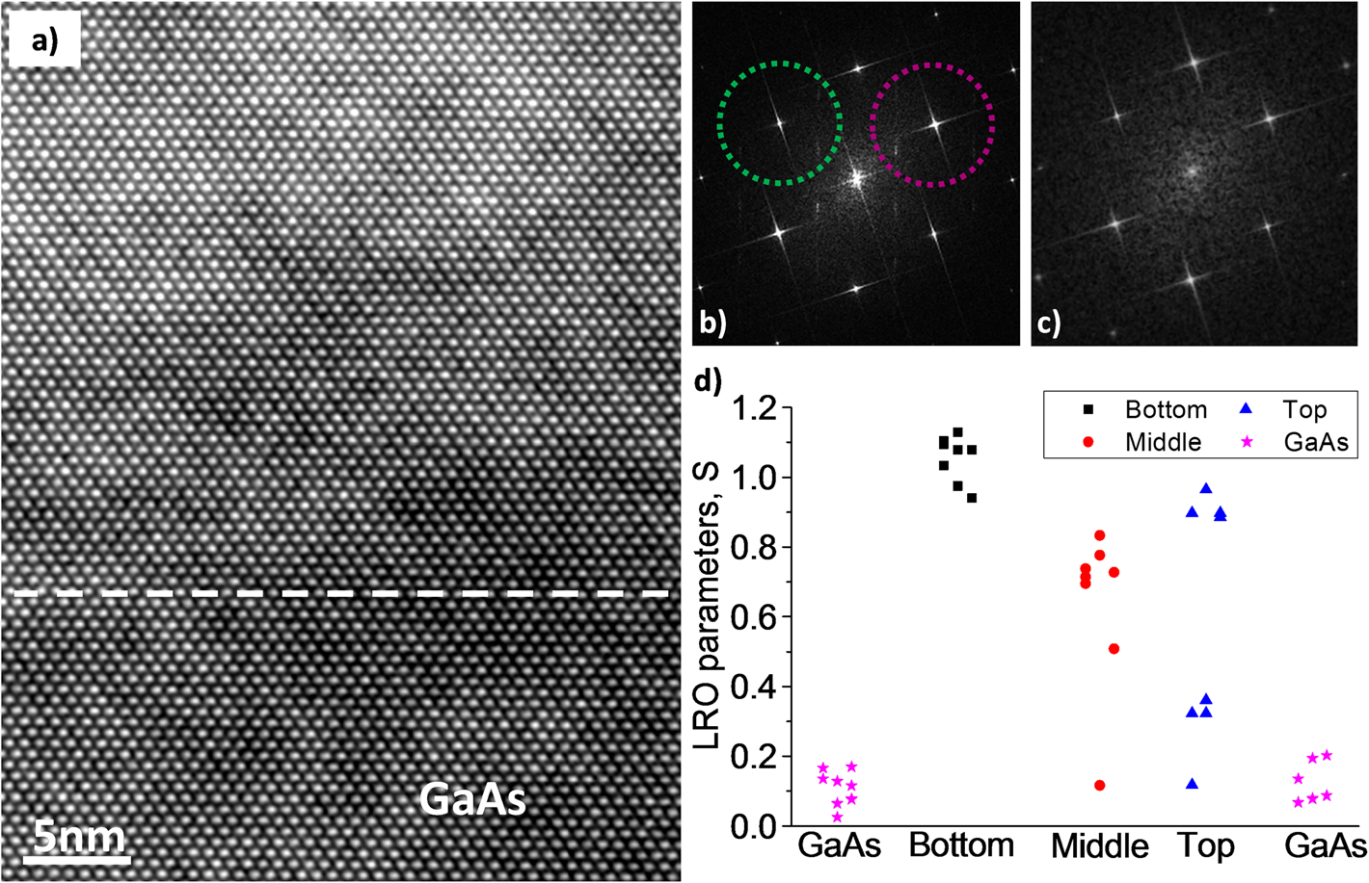
**Degree of ordering in sample S100. (a)** Cross-sectional HRTEM image taken along [110] at the lower interface of sample S100. The dashed line marks the interface between GaAs (below) and GaAsBi (above). **(b,c)** depict the FFT of (a) corresponding to GaAsBi area and GaAs, respectively. **(d)** The Bragg-Williams long-range order parameter (S) estimated along the layer of sample S100. The dashed circle mark the corresponding Bragg mask used to obtain the numerical moiré fringe maps of Figure 5.

Due to the difficulty in obtaining representative SAED patterns from the different regions of the GaAsBi layers, HRTEM images were acquired in the [110] zone axis in both samples to detect CuPt_B_-type ordering in the layers. Figure 4a displays an HRTEM image taken at the lower GaAs/GaAsBi interface of sample S100, and Figure 4b,c depicts the corresponding FFTs of the GaAsBi and GaAs regions of the image, respectively. The ½ 111-type spots in Figure 4b confirm the presence of CuPt_B_ ordering. This was also observed in sample S25, confirming the formation of CuPt_B_-type ordering that was too weak to be detected in the SAED pattern and highlighting the danger of relying on SAED analysis alone.

In order to obtain an estimate of how the ordering is distributed along the layer, we have analysed the intensity of ½ 111-type and 111-type spots in FFTs and calculated the order parameter from the bottom, middle and top of the layer in sample S100 (Figure 4d). The analysis revealed the absence of ordering within experimental error in the GaAs region (as expected) with an average LRO of 0.1, while the LRO was *S* ≅ 1 for both {111}_B_ families in the region closer to the bottom GaAs/GaAsBi interface (region I) in all HTREM images. Conversely, in the middle and top parts of the GaAsBi layers, regions both with and without ½ 111-type spots could be found and when present the LRO parameter varied between 0.3 and 1. It can therefore be concluded that there is a higher degree of ordering near the bottom interface.

### Ordering map

**Figure 5:**
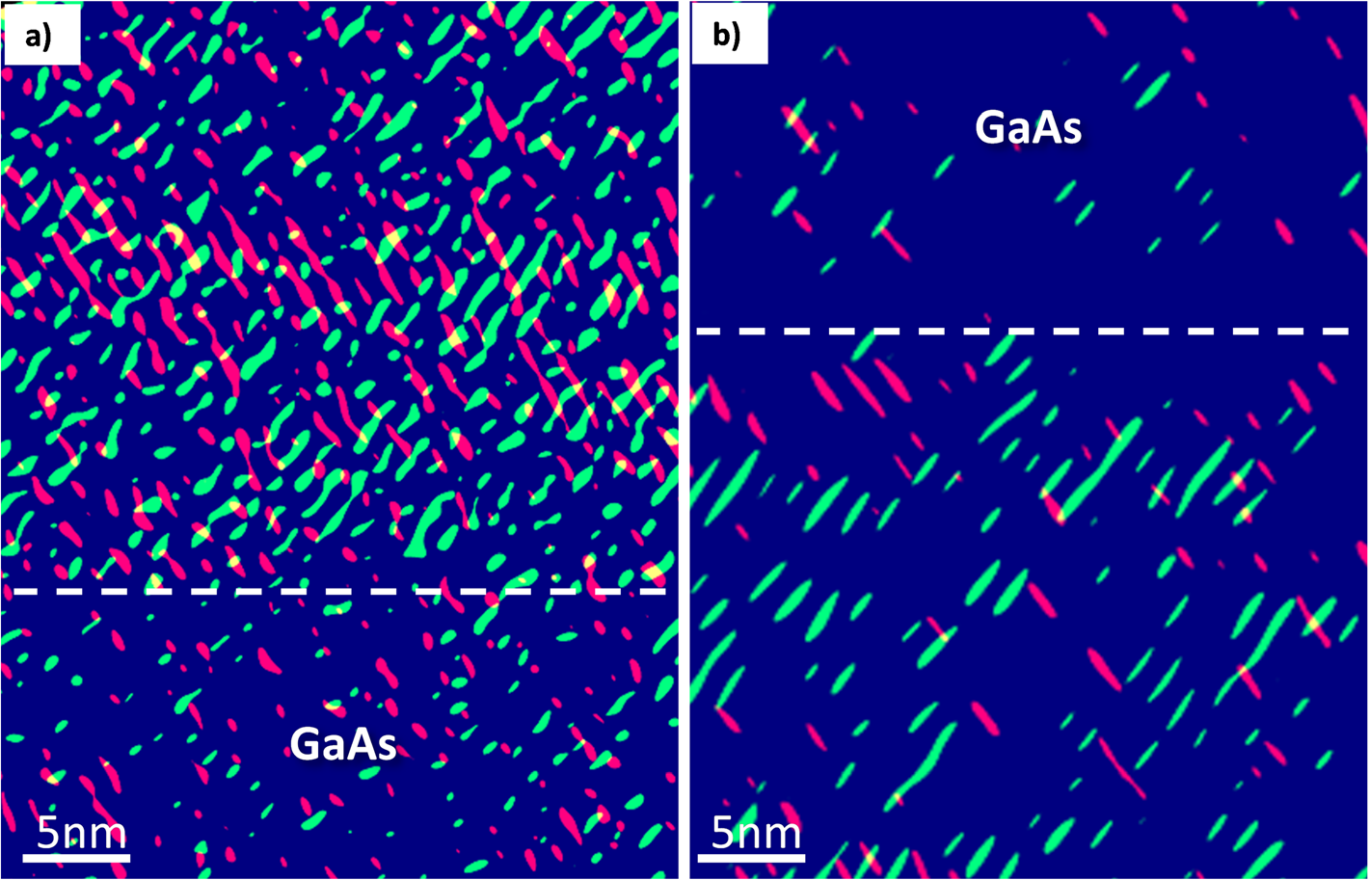
**Numerical moiré fringe maps obtained from HRTEM images.** The maps correspond to **(a)** region I (bottom) and **(b)** region II (top). Red and green fringes correspond to ordering on the two {111}_B_ planes. Dashed lines in **(a)** and **(b)** mark the beginning and end of the GaAsBi layer, respectively.

Figure 5 shows the ordering distribution map of the different regions of the GaAsBi layer obtained from HRTEM images. The RGB map reveals two different sets of lattice fringes (red and green) associated with the ordering observed in the two {111}_B_ planes. Intensity profiles plotted in the directions perpendicular to each set of moiré fringes (not shown here) depict a separation of 0.6 nm in between correlated fringes, changing the abcabc periodicity of crystal to a’bc’da’bc’d. The GaAs regions above and under the GaAsBi layers are shown for reference.

The ordering maps in region I show both variants coexisting in similar proportions over the whole GaAsBi layer. In addition, the estimated LRO parameters gave values of 1 for both {111}_B_ families. However, in region II of S100 with lower Bi content, the ordering is irregular, with lower LRO parameter (0.4 to 0.8) regions where one {111}_B_ family predominates and others where little ordering is present.

## Discussion

The ordering within the GaAs matrix is a phenomenon that occurs on {111} planes due to the distribution of atomic scale compressive and tensile strain sites. This distribution of solute atoms within the solvent matrix is believed to be responsible for enhanced solubility in GaAsBi [6] and GaInP [31]. However, growth of GaAsBi under a (2 × 1) reconstruction leads to anisotropic growth and a constantly increasing density of steps that eventually results in an undulating surface [9]. The undulations present compression (troughs) and tensile (peak) zones on the macroscopic scale. These macroscopic compressive and tensile zones occupying multiple near surface lattice sites offer a much more attractive strain relaxation centre compared to the individual atomic sites that lead to ordering. In S100, this switching point between preferred Bi incorporation sites leads to an evolution from CuPt_B_ ordering to phase separation at approximately 25 nm.

There is clearly a correlation between the degree of ordering and the Bi content, i.e. more ordering occurs in material with a higher Bi content. The CuPt ordered GaAsBi provides an attractive lattice site for Bi *in* the GaAs matrix. The undulation peaks offer attractive surface sites for Bi *on* a GaAs matrix, where a high local density of surface Bi exists on an undulation peak. Furthermore, the compressive troughs are highly unattractive surface occupancy sites for Bi. Thus, the overall Bi surface population is effectively halved and the Bi content of the GaAs matrix is subsequently reduced. The reduction in incorporation causes an excess of surface Bi and may result in Bi droplet formation. This would suggest that alloy clustering is only the favourable mechanism for Bi incorporation into the GaAs matrix when the growth surface is highly undulating. On a flat surface, before undulations occur, Bi incorporates from a homogeneous surface coverage in the form of Bi dimers on the (2 × 1) surface. Once incorporated into the surface, individual Bi atoms tend to move from nearest neighbours to next-nearest neighbours to minimize strain, thus generating atomic rows of alternating Bi and As [9]. Whilst this happens in a homogeneous manner on the flat surface leading to an *S* of 1, only the peaks contribute to this on the undulating surface, and hence, the ordering parameter is lower where the macroscopic distribution of the ordered Bi clusters corresponds to the period of the surface undulations present during growth. Thus, growth of thicker GaAsBi layers with homogeneous Bi content would require prevention of or restoration from the inherent undulating surface caused by the (2 × 1) reconstruction.

## Conclusions

In summary, we have analysed by optical and transmission electron microscopy techniques two GaAsBi layers grown by MBE with different thicknesses. Compositional analyses show that the bismuth content decreases exponentially in the first 25 nm from a maximum for both samples, followed by a region of almost constant Bi content in the thicker layer. This is consistent with the asymmetric shape of the PL emission peak in both cases, and the thicker layer behaves as a GaAsBi bilayer with two different compositions. CuPt_B_-type ordering is observed in SAED patterns and FFT analysis of HRTEM images. We have developed RGB multilayer maps showing the spatial locations of the two (111) ordering families in the layers. In addition, LRO parameter estimation from FFT intensities shows that ordering is almost complete in the lower region and diminishes in the upper region of the GaAsBi layers. A correlation between degree of ordering and dominant Bi incorporation mechanism is proposed.

## Competing interests

The authors declare that they have no competing interests.

## Authors’ contributions

FB designed and grew the sample and wrote the MBE growth sections. CH carried out the PL study and wrote the PL discussion section. JPRD supervised the PL analysis and interpretation of the energy transitions. DFR and AS acquired TEM data, carried out the analysed of results and drafted the manuscript. DG and DS designed the TEM studies, supervised the TEM analyses and participated in the draft of the manuscript. All authors read and approved the final manuscript.
